# Decolorization of a Corn Fiber Arabinoxylan Extract and Formulation of Biodegradable Films for Food Packaging

**DOI:** 10.3390/membranes11050321

**Published:** 2021-04-28

**Authors:** Verónica Weng, Carla Brazinha, Isabel M. Coelhoso, Vitor D. Alves

**Affiliations:** 1 LAQV-REQUIMTE, Department of Chemistry, NOVA School of Science and Technology, FCT NOVA, Universidade Nova de Lisboa, 2829-516 Caparica, Portugal; v.weng@campus.fct.unl.pt (V.W.); c.brazinha@fct.unl.pt (C.B.); imrc@fct.unl.pt (I.M.C.); 2 LEAF—Linking Landscape, Environment, Agriculture and Food, Instituto Superior de Agronomia, Universidade de Lisboa, Tapada da Ajuda, 1349-017 Lisboa, Portugal

**Keywords:** corn fiber, arabinoxylan, decolorization, biodegradable films, food packaging

## Abstract

Corn fiber from the corn starch industry is a by-product produced in large quantity that is mainly used in animal feed formulations, though it is still rich in valuable components, such as arabinoxylans, with proven film-forming ability. During arabinoxylans’ recovery under alkaline extraction, a dark-colored biopolymer fraction is obtained. In this work, a purified arabinoxylan extract from corn fiber with an intense brownish color was decolorized using hydrogen peroxide as the decolorizing agent. Biodegradable films prepared by casting the decolorized extract exhibited a light-yellow color, considered more appealing, envisaging their application in food packaging. Films were prepared with glycerol as plasticizer and citric acid as cross-linker. Although the cross-linking reaction was not effective, films presented antioxidant activity, a water vapor permeability similar to that of non-decolorized films, and other polysaccharides’ and mechanical properties that enable their application as packaging materials of low-water-content food products.

## 1. Introduction

Petroleum-based plastics are widely used and still play an important role as packaging materials and to produce a broad range of objects for our daily life, especially due to their unique properties and low production costs. There is, however, a rising concern in an environmental and ecological perspective since they are very resistant to degradation, including biodegradation, are often non-recyclable, and their burning as a way of disposing also releases toxic gases to the atmosphere, contributing to air pollution and global warming [1].

An approach to balance this high dependency on petroleum-based plastics is by exploring alternative ways, which can include the use of biodegradable polymers. According to the European Bioplastics Organization [1], biodegradable polymers may be bio-based, which include polymers extracted from biomass (polysaccharides and proteins), polymers produced by microorganisms (e.g., polyhydroxyalkanoates, polysaccharides), and polymers synthesized with microbial monomers (e.g., polylactic acid). In addition, there are also oil-based biodegradable polymers, such as polycaprolactone, poly(butylene succinate) (PBS) and its copolymers, and poly(butylene adipate-co-terephthalate) (PBAT) [2,3]. Though all mentioned polymer categories are usually referred to be biodegradable, only some of them, which include natural biopolymers from biomass-like polysaccharides, may be degraded by microorganisms under a wide range of environments (seawater, fresh water, soil, home and industrial composting, sewage sludge, and landfill) [4]. 

Polysaccharides are an important and diverse class in biopolymers, which can be found in great abundance in nature. Membranes produced from polysaccharides have good potential to be applied in the food industry as packaging or coatings. They are usually good barriers against gases like oxygen and carbon dioxide and have moderate mechanical properties. However, due to their hydrophilic nature, they show a low barrier against water vapor and a high water absorption and solubility in liquid water [3].

A wide range of polysaccharide-based membranes has been developed over the years, including the optimization of their functional properties to be used as packaging materials. Several strategies were developed, such as the addition of plasticizers, cross-linking agents, bioactive compounds, and lipids [5,6]. Some polysaccharides used include pectin, starch, cellulose, alginate, chitosan, and microbial polysaccharides (FucoPol) [3,7]. As examples, composite pectin-cocoa butter films were prepared with glycerol as a plasticizer, presenting the potential for food applications [8]. Films of starch from cassava residues, reinforced with modified cellulose nanocrystals, and glycerol as a plasticizer were also produced [9]. Antioxidative and antimicrobial chitosan films were obtained with the incorporation of leaf, stem, and seed extract from *Pistacia terebinthus* and glycerol as a plasticizer [10]. Films from FucoPol produced with *Enterobacter A47* were obtained using citric acid as cross-linker [11]. Chitosan with different nanofillers enhanced the barrier and mechanical properties of the films for food packaging [12].

Food processing industries result in several low-valued by-products, which are usually considered as waste or used as animal feed. However, most of these residues still contain in their composition compounds with interesting properties [13,14]. An example of these by-products is corn fiber from the corn starch industry that is mainly incorporated in animal feed. However, it is composed of ferulic acid, a phenolic compound with antioxidant activity and arabinoxylan with film-forming properties. Therefore, there is interest in the valorization of both components [13,15].

Arabinoxylans are polysaccharides found in cereal grains like corn/maize, wheat, oats, barley, rice, sorghum, and rye [16,17]. They are constituted by a linear backbone of *β*-(1,4)-xylopyranosyl, which can be substituted with *α*-L-arabinofuranosyl residues in both O-3 and O-2 positions, only O-3, only O-2, or simply not have any substituents. These arabinose substituents can then be found esterified in O-5 with ferulic acid. The amount and distribution of arabinose residues, as well as the content of ferulic acid in the polysaccharide, vary with the origin and extraction method. Additionally, arabinoxylans can be classified according to their solubility in water as water-extractable and water-unextractable. Due to cross-linking reactions between arabinoxylan and cell wall components, a portion of this polysaccharide is water insoluble. Therefore, to recover these fractions, usually, an alkaline extraction is applied to hydrolyze these linkages [18].

Purification methods are important to remove contaminants and obtain an extract rich in arabinoxylans. Some reported purification methods for arabinoxylan extracts include membrane processes, such as diafiltration in a hollow-fiber membrane [15] or dialysis, to remove low-molecular-weight contaminants [19].

In a previous work, a fraction enriched in arabinoxylans obtained by alkaline extraction from corn fiber was purified using membrane processes, and the obtained arabinoxylans were used to produce stand-alone films [15]. However, the films obtained were not transparent, presenting a brownish color, which may not be appealing if they are intended for food packaging. As such, in the present work, two different decolorization methods were explored to remove the color of the purified arabinoxylan extract, before using the polysaccharide fraction for films’ production. In addition to that, citric acid was studied as cross-linking agent to enhance films’ moisture resistance. Films were characterized in terms of color, solubility in water, water vapor permeability, mechanical properties, and antioxidant activity.

## 2. Materials and Methods

### 2.1. Materials

Arabinoxylan was obtained from alkaline extraction of corn fiber (with an aqueous solution of 0.25 M of sodium hydroxide, under stirring at 30 °C for 7 h), and the extract was purified in a hollow-fiber membrane unit (UFP-100-C-5A, from GE Healthcare, Chicago, IL, USA), according to the procedure used by Serra et al., 2020 [15]. The purified extract was freeze-dried, and the solid was stored in a sealed plastic bag at −20 °C. Activated charcoal (Sigma-Aldrich, St. Louis, MO, USA) and hydrogen peroxide (José Manuel Gomes dos Santos LDA, Odivelas, Portugal) were used for the decolorization process. For the formulation of films, glycerol (Sigma-Aldrich, USA) and citric acid (PanReac, Barcelona, Spain) were used. For antioxidant activity determination, sodium acetate (Riedel-de-Haën, Seelze, Germany), acetic acid glacial (Fisher Chemical, Loughborough, UK), TPTZ (2,3,5-triphenyltetrazolium chloride) (Sigma-Aldrich, USA), HCl (Honeywell, Wien, Austria), and ferric chloride (PanReac, Spain) were used.

### 2.2. Decolorization Process

The decolorization of purified arabinoxylan extract was tested using two methods: adsorption by activated charcoal and reaction with hydrogen peroxide. In the first case, freeze-dried extract was used to prepare an arabinoxylan solution (2% *w*/*v*) with deionized water to which activated charcoal was added (0.5% or 2% *w*/*v*). The mixture was stirred for 1 h at room temperature and then filtered. Absorption spectra were measured before and after contact with activated charcoal using a spectrophotometer (Evolution 201, Thermo Scientific, Waltham, MA, USA). 

When using hydrogen peroxide, the arabinoxylan solution (2% *w*/*v*) was firstly heated up to 45 °C and hydrogen peroxide (10% *v*/*v*_final_) was added. The mixture was left at 45 °C for 2 h under stirring and absorption spectra were measured before and after the reaction. 

### 2.3. Preparation of Films

Films without decolorization were prepared by dissolving freeze-dried arabinoxylan (Ax) in deionized water (2% *w*/*v*), to which glycerol 30% (*w*/*w*_Ax basis_) was added as plasticizer. The mixture was stirred for 15 min and, afterwards, a volume of 10 mL was cast in 50-mm plastic Petri dishes. The Petri dishes were left for 24 h at 35 °C and at a relative humidity of 30% for solvent drying. The films were peeled and stored in a desiccator at room temperature with a relative humidity of 62%, until further characterization.

Films with decolorization were produced using the resulting solution from the decolorization method using hydrogen peroxide (10% *v*/*v*_final_). To this solution, glycerol 30% (*w*/*w*_Ax basis_) and citric acid 10% (*w*/*w*_Ax basis_) were also added, as plasticizer and cross-linking agents, respectively. The final mixture was cast and dried and the films were stored, as described previously. The effect of thermal treatment (90 °C, 1 h) on the cross-linking reactions was also studied for decolorized films. Overall, five different films were developed according to Table 1.

### 2.4. Films’ Characterization

#### 2.4.1. Color Measurement

The color was measured in triplicate using a colorimeter (Chroma Meter CR-300, Minolta, Tokyo, Japan), using the CIELAB or CIE L* a* b* color system. The colorimeter was calibrated against a white standard where L* = 97.21, a* = 0.14, and b* = 1.99, with L* being lightness, a* and b* being the chromaticity coordinates: a* from red (positive) to green (negative) and b* from yellow (positive) to blue (negative). Hue (*h*°), which represents the angle on the chromaticity axis, was calculated using Equations (1) and (2):

(1) $$h^{{}^{\circ}}=arctan\frac{b^{*}}{a^{*}}\times \frac{180}{\pi }\;for\;a^{*}\;and\;b^{*}>0$$

or

(2) $$h^{{}^{\circ}}=\left(arctan\frac{b^{*}}{a^{*}}\times \frac{180}{\pi }\right)+180,\;for\;a^{*}<0$$

Chroma (C*) or saturation of color was calculated by Equation (3),

(3) $$C^{*}={\left({\left(a^{*}\right)}^{2}+{\left(b^{*}\right)}^{2}\right)}^{\frac{1}{2}}$$

#### 2.4.2. Antioxidant Activity by Ferric Reduction Antioxidant Power (FRAP) Method

The antioxidant activity of films was determined by the FRAP method in triplicate. FRAP reagent was prepared with 25 mL of acetate buffer 0.3 M, pH = 3.6, 2.5 mL of TPTZ (2,3,5-triphenyltetrazolium chloride) solution 10 mM in HCl 40 mM, and 2.5 mL of ferric chloride 20 mM. In a test tube, 270 μL of deionized water, 2.7 mL of FRAP reagent, and around 2 mg of film sample were added. The mixture was homogenized in a vortex (RSLAB-6PRO, Auxilab S.L., Navarra, Spain) and incubated at 37 °C in a water bath (Precision, Thermo Scientific, USA) for 30 min. Then, the solutions were diluted in a 1:3 proportion and the absorbance was measured at λ = 595nm (Cary 100 UV-Vis, Agilent Technologies, Santa Clara, CA, USA). A standard curve of absorbance vs. Trolox concentration was used to determine the antioxidant activity (in Trolox equivalents).

#### 2.4.3. Solubility

Film solubility was determined following Ferreira et al., 2016 [20] method, with slight modifications. Samples (10 × 10 mm) were cut in triplicates and dried in an oven at 50 °C for 24 h. After this time, samples were weighed to obtain the initial dry mass (*m*_1_, g), then immersed in 5 mL of deionized water under orbital stirring (Mistral Multi-Mixer, Lab-Line Instruments, Inc., Melrose Park, IL, USA) for 24 h. The resulting solution was centrifugated at 5000 rpm for 5 min and the liquid was discarded. The solid residue was dried at 50 °C for 24 h and weighed to obtain the final mass (*m*_2_, g). Solubility was calculated using Equation (4):

(4) $$S\;\left(\%\right)=\frac{m_{1}-m_{2}}{m_{1}}\times 100$$

#### 2.4.4. Mechanical Tests 

Perforation tests were carried in a TA-XT plus texturometer (Stable Micro Systems, Surrey, UK). Samples were fixed in a support with a 1-cm-diameter hole in the middle. To measure the force necessary to perforate the sample, we used a cylindrical probe with 2 mm of diameter, travelling at a constant velocity of 1 mm/sec. The tension of perforation (σ_p_, Pa) was calculated as the ratio between the perforation force (*F*_P_, N) and the circular area of the probe (*A*_c_, m^2^). To calculate the film sample deformation during the test, we considered the radius of the support hole (initial length) as the adjacent side of a triangle rectangle and the distance travelled by the probe until the moment the film was perforated as the opposite side. The stretching length was then obtained as the hypotenuse. Deformation was calculated as the difference between the stretching length and the initial length divided by the initial length. Three film replicates were analyzed.

#### 2.4.5. Films’ thickness and Water Vapor Permeability

Films’ thickness was measured using a digital micrometer (Digimatic Micrometer, Mitutoyo, Kawasaki, Japan). Water vapor permeability was determined gravimetrically following the method by Ferreira et al., 2016, and Serra et al., 2020 [15,20] with slight modifications. Circular film samples (diameter of 3 cm) were sealed with aluminum tape on top of glass cups containing a saturated solution of magnesium nitrate (*a*_w_ = 0.529). These glass cups were then placed in a desiccator containing a saturated solution of magnesium chloride (*a*_w_ = 0.328), which was equipped with a fan to promote air circulation and to minimize the resistance of mass transfer above the film. Relative humidity and temperature were measured with a thermohygrometer (HUMICAP® HM40, Vaisala, Helsinki, Finland) in regular time intervals, as well as the weight of the cups, for 8 h. Water vapor permeability was then calculated with Equation (5):

(5) $$WVP=\frac{N_{w}\times \delta }{\Delta P_{w\cdot eff}}$$

where *N*_w_ (mol/m^2^·s) is the water vapor flux, δ (m) is the thickness of the film, and ∆*P*_w·eff_ (Pa) is the effective driving force, which was estimated according to the method used by Alves et al., 2010 [21]. Three film replicates were analyzed.

#### 2.4.6. Statistical Analysis

Statistica 7.0 software (Statsoft Inc., Tulsa, OK, USA) was used to perform analysis of variance and post hoc Scheffe test (*p* level of 0.05) was used in order to detect differences among mean values of films’ properties.

## 3. Results and Discussion

### 3.1. Decolorization of Arabinoxylan Extract

#### 3.1.1. Adsorption by Activated Charcoal Method

**Figure 1:**
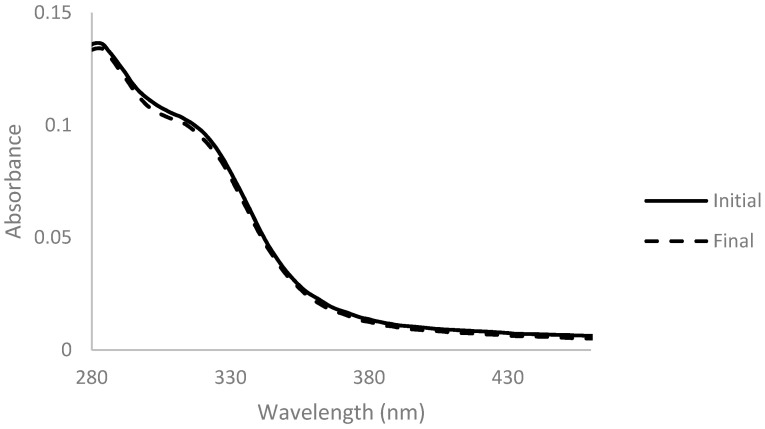
Absorbance spectra of arabinoxylan extract with 0.5% (*w*/*v*) of activated charcoal before (full line) and after 1 h of contact (dashed line), for a dilution ratio of 1:200.

**Figure 2:**
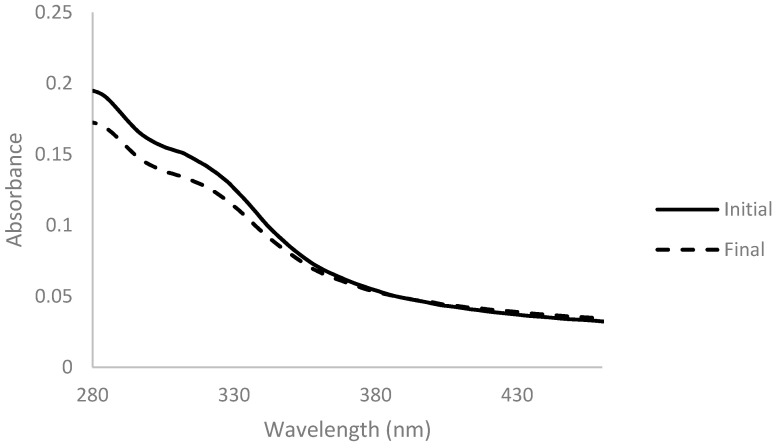
Absorbance spectra of arabinoxylan extract with 2% (*w*/*v*) of activated charcoal before (full line) and after 1 h of contact (dashed line), for a dilution ratio of 1:200.

Arabinoxylan was decolorized with activated charcoal at two different concentrations of 0.5% and 2% (*w*_activated charcoal_/*v*_solution_) for 1 h at room temperature. Absorbance spectra were measured before and after the addition of activated charcoal and are presented below (Figure 1 and Figure 2).

**Figure 3:**
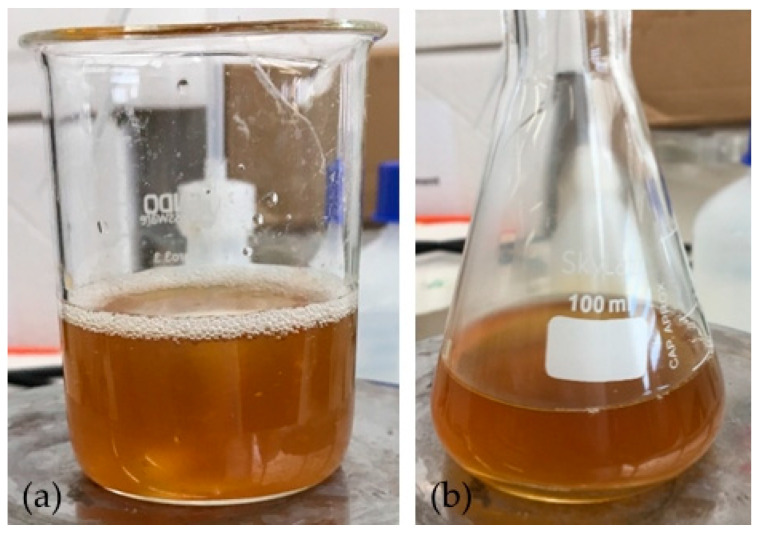
Solution of arabinoxylan (2% *w*/*v*): (**a**) before the addition of activated charcoal (2% *w*/*v*) and (**b**) after 1 h of contact.

The spectrum obtained with 2% activated charcoal showed a more accentuated decrease in absorbance after the addition when compared with the spectrum with only 0.5%. This change indicates that the activated charcoal was able to adsorb components present in the solution to some extent. However, the color of the solution did not change when observed at naked eye in both cases (Figure 3).

#### 3.1.2. Hydrogen Peroxide Method

**Figure 4:**
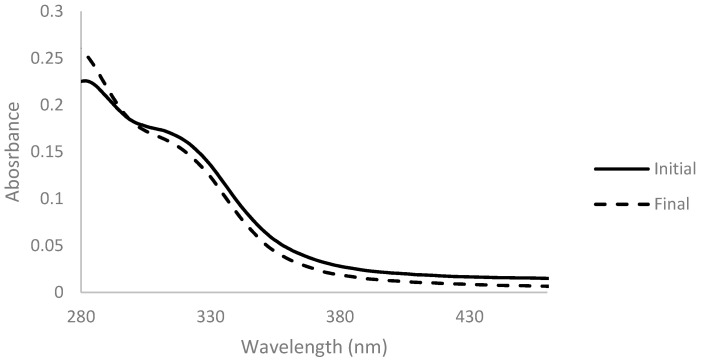
Absorbance spectra of arabinoxylan 2% (*w*/*v*) with 10% (*v*/*v*_final_) of hydrogen peroxide before (full line) and after 2 h of reaction time at 45 °C (dashed line), for a dilution ratio of 1:100.

Another method was tested for the decolorization of arabinoxylan, employing hydrogen peroxide. A solution of 2% (*w*/*v*) of arabinoxylan was prepared, and hydrogen peroxide in a concentration of 10% (*v*/*v*_final_) was added. Absorbance spectra were measured before and after the addition and are presented below (Figure 4).

**Figure 5:**
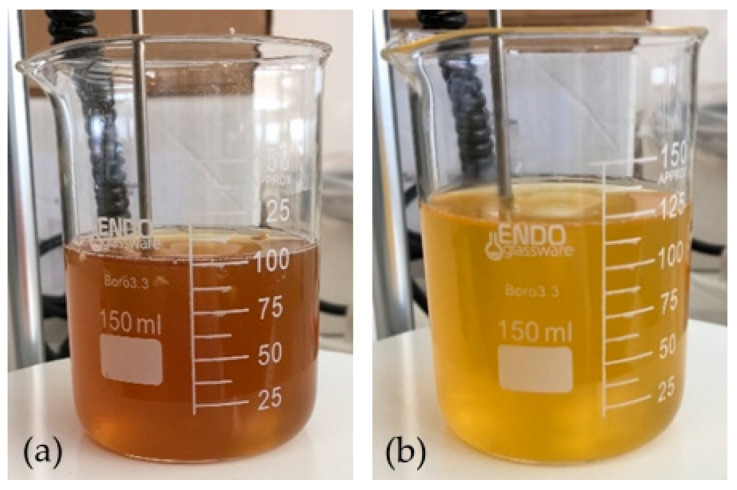
Solution of arabinoxylan (2% *w*/*v*): (**a**) before the addition of hydrogen peroxide (10% *v*/*v*_final_) and (**b**) after 2 h at 45 °C of reaction time.

A lower absorbance of the solution in the 300-nm-onwards zone of the spectrum after reaction was noticed. This change was complemented with a change of the color of the solution, which shifted from a darker and brownish color to a lighter and yellow color (Figure 5). 

Additionally, a higher hydrogen peroxide concentration (20% *v*/*v*_final_) and reaction time (5.5 h) were also tested. However, no significant improvement in the color of the solution was noted. Therefore, considering the results obtained above, the decolorization process continued with the hydrogen peroxide method as described in Section 2.2.

### 3.2. Films’ Characterization

#### 3.2.1. Color Measurement

The color of films was measured, as described in Section 2.4.1, and the results are summarized in Table 2.

**Figure 6:**
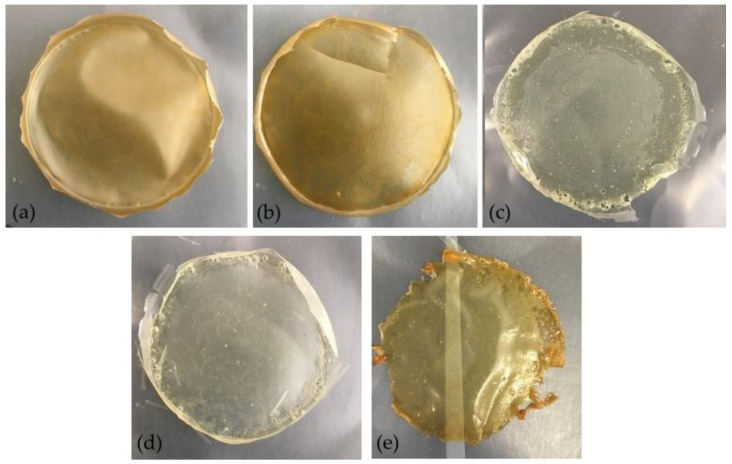
Films based on arabinoxylan (Ax): (**a**) 1 (Ax), (**b**) 2 (Ax + Glycerol), (**c**) 3 (Ax + Glycerol + H_2_O_2_), (**d**) 4 (Ax + Glycerol + H_2_O_2_ + Citric Acid), and (**e**) 5 (Ax + Glycerol + H_2_O_2_ + Citric Acid + 90 °C, 1 h).

The hue values calculated, around 89° and 100° for non-decolorized and decolorized films, respectively, indicated decolorized films presenting a color closer to a light-yellow, and films without decolorization a tonality close to dark yellow-orange, consistent with the images of Figure 6. 

The non-decolorized arabinoxylan films prepared in a previous work [15], with 30% glycerol (*w*/*w*_dry basis_), presented hue values (*h*° = 86.64 ± 1.28) similar to those of this work. In addition to hue, color saturation (C*) also changed substantially when applying the decolorization method, decreasing to values around 9 and 15. The results showed that, even though full transparency was not obtained when applying decolorization, a more pleasant light-yellow color could be achieved.

The film that was subjected to heat treatment presented color values in between the two mentioned above. Since it did not bring positive results in terms of color, this method was discarded.

#### 3.2.2. Antioxidant Activity

Antioxidant activity was determined by the FRAP method, by dissolving film samples directly in the FRAP reagent. Results are presented below (Table 3). 

Films were expected to show antioxidant activity due to the presence of ferulic acid and other molecules, which were released during the arabinoxylan alkaline extraction from corn fiber and remained in the extract after its purification with membrane processes [13,15].

According to the results, the addition of other components than arabinoxylan while preparing the filmogenic solutions appeared to slightly reduce the antioxidant activity of the resulting films. The major decrease was noticed when the films were produced from decolorized purified extract. This fact may be attributed to the antioxidant’s reaction with H_2_O_2,_ applied with the intention of removing colored substances. An arabinoxylan film previously prepared in a previous work with the same formulation as 2 (Arabinoxylan with 30% *w*/*w*_Ax basis_ of glycerol) showed an antioxidant activity of (1.56 ± 0.85) × 10^−5^ mmol Trolox/mg film [15]. All films prepared in this work showed a higher value, which can be due to differences in the composition of the purified arabinoxylan among the different batches produced, more specifically regarding the quantity of ferulic acid present.

#### 3.2.3. Films’ Cross-Linking and Solubility in Water

The values of films’ solubility in water are presented in Table 4.

It can be observed that all films were highly soluble in water. It appears that the decolorization process did not significantly change the hydrophilic nature of arabinoxylan polymeric matrix. In addition, the performance of citric acid as a cross-linker under the conditions tested was not effective, as the solubility in water of cross-linked and non-cross-linked films was not significantly different. The cross-linking reaction expected was esterification, which is not very favorable in an aqueous medium [22]. However, due to the nature of the extract (insoluble in most solvents), it was difficult to carry the reaction in different media besides water. When comparing with other polysaccharides, FucoPol and chitosan films presented a quite lower solubility in water (47.5 ± 5.2% and 30.5 ± 0.5%, respectively) [20] than the produced films in this work.

#### 3.2.4. Water Vapor Permeability

The water vapor permeability values, determined by the gravimetric method described in Section 2.4.5, are presented in Table 5. 

Due to the hydrophilic nature of arabinoxylan, we expected a low water vapor barrier of the tested films. The addition of glycerol or any plasticizer is usually followed by an increase of WVP since these molecules incorporated in the polysaccharide matrix decrease the molecular density and consequently increase the water vapor diffusion. However, in this work, the glycerol addition was not reflected significantly in the results obtained, since the WVP values were quite similar between the different film formulations.

When comparing with an arabinoxylan film with 15% (*w*/*w*_Ax basis_) of glycerol [23], with a value of WVP = (0.98 ± 0.03) × 10^−11^ mol·m/m^2^·s·Pa (driving force 84–22%, RH(%)), it may be seen that the produced film in the present work with a higher content of glycerol (Ax + Glycerol 30% *w*/*w*_Ax basis_) did have a higher value of water vapor permeability. However, when compared with another arabinoxylan film prepared in a different work, with the same glycerol content (WVP = (0.62 ± 0.02) × 10^−11^ mol·m/m^2^·s·Pa, driving force 52.9–32.8%, RH(%)) (5), the equivalent film prepared in this work had a higher WVP value (WVP=(2.62 ± 0.96) × 10^−11^ mol·m/m^2^·s·Pa). This behavior can be explained by differences in the composition of the arabinoxylan extract, purification methods, and casting and drying conditions that alter the state of the resulting film.

Comparing values of water vapor permeability of other polysaccharide films like FucoPol (WVP = (0.75 ± 0.05) × 10^−11^ mol·m/m^2^·s·Pa, driving force 76.9–22.5%, RH(%)), it may be seen that the present films show a higher permeability. However, when compared with films from chitosan (WVP = (4.13 ± 0.13) × 10^−11^ mol·m/m^2^·s·Pa, driving force 76.9–22.5%, RH(%)), some of the obtained films in this work showed a lower water vapor permeability. It is also important to mention that WVP is highly dependent on the driving force used, in addition to the other variables mentioned above [20].

#### 3.2.5. Mechanical Tests 

Perforation tests were carried out in quadruplicate and the mechanical parameters measured are presented in Table 6. 

The film sample produced only with arabinoxylan (Ax) presented a brittle, non-ductile structure evidenced by the rather low deformation upon perforation (1.3 ± 0.3%). The need for using a plasticizer is evidenced by the higher mechanical resistance achieved when glycerol was added to the formulation (Ax + Glycerol), with a substantial increase of tension and deformation upon perforation (2.36 ± 0.3 MPa and 50.8 ± 4.8%, respectively). An arabinoxylan film from maize bran with 25% of glycerol in its composition showed a value tension of perforation of 2.42 ± 0.12 MPa [16], which is very similar to that of the prepared film in this work with 30% of glycerol. 

The addition of H_2_O_2_ for decolorization led to films with lower tension of perforation, while maintaining a substantial deformation (41.3 ± 3.1% and 53.0 ± 1.7% for films 3 and 4, respectively). As such, the results envisage the maintenance of suitable mechanical properties for decolorized film samples. 

## 4. Conclusions

The decolorization of a purified arabinoxylan extract from corn fiber was achieved using hydrogen peroxide as decolorizing agent. This process originated a more appealing light-yellow solution that retained its film-forming properties. Decolorized films of arabinoxylan were prepared with glycerol as plasticizer and citric acid as cross-linker. Though the cross-linking reaction was not successful, since the film incorporated with citric acid still presented a high solubility in water, films showed promising properties. Decolorized films with glycerol and citric acid retained a significant antioxidant activity, and values of water vapor permeability were similar to that of non-decolorized ones and other polysaccharides. In addition, the decolorization process did not affect substantially the films’ mechanical properties under perforation tests. 

New strategies are necessary to improve the conditions for cross-linking reactions, by either changing the solvent or choosing a different cross-linker. To improve the properties of resulting films, arabinoxylan blends with other polysaccharides or biopolymers with hydrophobic nature are envisaged.

## Figures and Tables

**Table 1:** Different conditions tested in the production of arabinoxylan (Ax) films, with decolorization process employing hydrogen peroxide, glycerol as plasticizer, citric acid as cross-linker, and heat treatment at 90 °C.

Film	Ax (2% *w*/*v*)	Decolorized (H_2_O_2_)	Glycerol (30% *w*/*w*_Ax basis_)	Citric Acid (10% *w*/*w*_Ax basis_)	Heat Treatment (90 °C, 1 h)
1	X				
2	X		X		
3	X	X	X		
4	X	X	X	X	
5	X	X	X	X	X

**Table 2:** Values of lightness (L*), chromaticity coordinates (a*, b*), and the calculated hue (*h*°) and chroma (C*) for the arabinoxylan films obtained. Values in the same column followed by different superscript letters differ significantly (*p* < 0.05).

Film	L*	a*	b*	*h*°	C*
1 (Ax)	80.29 ± 0.69 ^a^	0.65 ± 0.32 ^a^	43.60 ± 1.21 ^a^	89.15 ± 0.40 ^a^	43.60 ± 1.21 ^a^
2 (Ax + Glycerol)	79.65 ± 0.99 ^a^	0.56 ± 0.48 ^a^	46.85 ± 1.90 ^b^	89.33 ± 0.57 ^a^	46.86 ± 1.91 ^b^
3 (Ax + H_2_O_2_+Glycerol)	95.64 ± 0.24 ^b^	−1.77 ± 0.17 ^b^	8.69 ± 0.64 ^c^	101.48 ± 0.23 ^b^	8.87 ± 0.66 ^c^
4 (Ax + H_2_O_2_ + Glycerol + Citric Acid)	94.94 ± 0.23 ^b^	−2.62 ± 0.19 ^c^	14.56 ± 1.00 ^d^	100.19 ± 0.24 ^b^	14.79 ± 1.01 ^d^
5 (Ax + H_2_O_2_ + Glycerol + Citric Acid + 90 °C, 1 h)	89.03 ± 0.88 ^c^	−1.55 ± 0.10 ^b^	28.04 ± 1.00 ^e^	93.16 ± 0.29 ^c^	28.08 ± 0.99 ^e^

**Table 3:** Antioxidant activity of films obtained expressed as Trolox equivalents (mmol Trolox/mg film). Values in the same column followed by different superscript letters differ significantly (*p* < 0.05).

Film	Antioxidant Activity (10^−5^ mmol Trolox/mg Film)
1 (Ax)	5.09 ± 0.14 ^a^
2 (Ax + Glycerol)	4.15 ± 0.46 ^b^
3 (Ax + H_2_O_2_ + Glycerol)	3.53 ± 0.39 ^b,c^
4 (Ax + H_2_O_2_ + Glycerol + Citric Acid)	3.21 ± 0.40 ^c^

**Table 4:** Solubility in water (%) of arabinoxylan films obtained. Values in the same column followed by different superscript letters differ significantly (*p* < 0.05).

Film	Solubility (%)
1 (Ax)	86 ± 10 ^a^
2 (Ax + Glycerol)	87 ± 10 ^a^
3 (Ax + H_2_O_2_ + Glycerol)	70 ± 13 ^a^
4 (Ax + H_2_O_2_ + Glycerol + Citric Acid)	82 ± 10 ^a^

**Table 5:** Water vapor permeability (WVP) (10^−11^ mol·m/m^2^·s·Pa) and thickness (μm) of arabinoxylan films tested. Values in the same column followed by different superscript letters differ significantly (*p* < 0.05).

Film	Thickness (μm)	WVP (10^−11^ mol·m/m^2^·s·Pa)
1 (Ax)	76 ± 6	3.89 ± 1.99 ^a^
2 (Ax + Glycerol)	101 ± 12	2.62 ± 0.96 ^a^
3 (Ax + H_2_O_2_+ Glycerol)	79 ± 2	3.46 ± 0.13 ^a^
4 (Ax + H_2_O_2_ + Glycerol + Citric Acid)	94 ± 3	2.94 ± 0.49 ^a^

**Table 6:** Tension of perforation (MPa), deformation (%), and thickness (μm) of arabinoxylan films. Values in the same column followed by different superscript letters differ significantly (*p* < 0.05).

Film	Thickness (μm)	Tension of Perforation (MPa)	Deformation (%)
1 (Ax)	79 ± 6	1.17 ± 0.23 ^a^	1.3 ± 0.3 ^a^
2 (Ax + Glycerol)	84 ± 4	2.36 ± 0.30 ^b^	50.8 ± 4.8 ^b^
3 (Ax + H_2_O_2_ + Glycerol)	89 ± 11	1.57 ± 0.15 ^a^	41.3 ± 3.1 ^c^
4 (Ax+H_2_O_2_+ Glycerol + Citric Acid	105 ± 19	1.22 ± 0.41 ^a^	53.0 ± 1.7 ^b^

## Data Availability

Not applicable.
